# The prevalence, antibiotic resistance and *mecA* characterization of coagulase negative staphylococci recovered from non-healthcare settings in London, UK

**DOI:** 10.1186/s13756-018-0367-4

**Published:** 2018-06-13

**Authors:** Zhen Xu, Haroun N. Shah, Raju Misra, Jiazhen Chen, Wenhong Zhang, Yuting Liu, Ronald R. Cutler, Hermine V. Mkrtchyan

**Affiliations:** 1 National Demonstration Center for Experimental Preventive Medicine Education, Tianjin Medical University, Qixiang Road No. 22, Tianjin, 300070 China; 2 School of Biological and Chemical Sciences, Queen Mary University of London, London, UK; 3 Department of Natural Sciences, Middlesex University, The Burroughs Hendon, Middlesex, NW4 4BT UK; 4 Natural History Museum, Cromwell Rd, London, SW7 5BD UK; 5 Department of Infectious Diseases, Huashan Hospital, Fudan University, Shanghai, 200040 China; 6 Tianjin Xiqing hospital, Tianjin, 300380 China; 7 School of Health, Sport and Biosciences, University of East London, E1 4NS, London, UK

**Keywords:** CoNS, Antibiotic resistance, SCC*mec*, MLST

## Abstract

**Background:**

Coagulase negative staphylococci (CoNS) are important reservoirs of antibiotic resistance genes and associated mobile genetic elements and are believed to contribute to the emergence of successful methicillin resistant *Staphylococcus aureus* (MRSA) clones. Although, these bacteria have been linked to various ecological niches, little is known about the dissemination and genetic diversity of antibiotic resistant CoNS in general public settings.

**Methods:**

Four hundred seventy-nine samples were collected from different non-healthcare/general public settings in various locations (*n* = 355) and from the hands of volunteers (*n* = 124) in London UK between April 2013 and Nov 2014.

**Results:**

Six hundred forty-three staphylococcal isolates belonging to 19 staphylococcal species were identified. Five hundred seventy-two (94%) isolates were resistant to at least one antibiotic, and only 34 isolates were fully susceptible. Sixty-eight (11%) *mecA* positive staphylococcal isolates were determined in this study. SCC*mec* types were fully determined for forty-six isolates. Thirteen staphylococci (19%) carried SCC*mec* V, followed by 8 isolates carrying SCC*mec* type I (2%), 5 SCC*mec* type IV (7%), 4 SCC*mec* type II (6%), 1 SCC*mec* type III (2%), 1 SCC*mec* type VI (2%), and 1 SCC*mec* type VIII (2%). In addition, three isolates harboured a new SCC*mec* type 1A, which carried combination of class A *mec* complex and *ccr* type 1.

MLST typing revealed that all *S. epidermidis* strains possess new MLST types and were assigned the following new sequence types: ST599, ST600, ST600, ST600, ST601, ST602, ST602, ST603, ST604, ST605, ST606, ST607 and ST608.

**Conclusions:**

The prevalence of antibiotic resistant staphylococci in general public settings demonstrates that antibiotics in the natural environments contribute to the selection of antibiotic resistant microorganisms. The finding of various SCC*mec* types in non-healthcare associated environments indicates the complexity of SCC*mec*. We also report on new MLST types that were assigned for all *S. epidermidis* isolates, which demonstrates the genetic variability of these isolates.

**Electronic supplementary material:**

The online version of this article (10.1186/s13756-018-0367-4) contains supplementary material, which is available to authorized users.

## Background

Staphylococci are the most frequently isolated nosocomial pathogens, accounting for 30% of hospital associated infections [1]. Despite, that the high virulence of *S. aureus* has been evidenced in many studies [2], it is believed that coagulase-negative staphylococci (CoNS) act as an important reservoir of antimicrobial resistance genes and resistance-associated mobile genetic elements, which can transfer between staphylococcal species. Among other CoNS, *S. epidermidis, S. hominis* and *S. haemolyticus* are often reported to be resistant to multiple antibiotics [3, 4].

The *mecA* gene responsible for methicillin resistance was first determined in *S. aureus*, however, many other staphylococcal species were found to also harbour it [5]. The *mecA* gene encodes an additional penicillin-binding protein 2a (PBP 2a), which mediates cell wall synthesis in the presence of β-lactam antibiotics [6]. Together with its regulators *mecI-mecR1* and site specific recombination genes *ccrA* and *ccrB*, the *mecA* gene, is located on a mobile genetic element known as staphylococcal cassette chromosome *mec* (SCC*mec*) [7]. A number of studies have demonstrated the transfer of *mecA* gene from coagulase-negative staphylococcal species to *S. aureus* in vivo, and thus contributing to more successful *S. aureus* clones [8]. To date 11 SCC*mec* types have been reported based on combinations of *mec* (A, B, C1, C2 and D) and *ccr* (AB1, AB2, AB3, AB4 and *ccrC*) complexes and so called J regions (1, 2, 3) [9].

Traditionally recognised as hospital associated pathogens, methicillin resistant coagulase negative staphylococci (MR-CoNS) have recently been linked with a range of ecological niches (community, wildlife and environmental sources) [10–12]. As a result, today increasing attention is being paid to the rapid spread of MR-CoNS and their role in transmission within the community and non - hospital settings [13].

In this study we demonstrate the dissemination of antibiotic resistance in CoNS isolated from various environmental sites in London, UK. The characterization of *mecA* gene and the SCC*mec* elements provide insights into the diversity of environmental CoNS clones.

## Methods

### Isolation

Four hundred seventy-nine samples were collected from different environmental sites in various locations (*n* = 355) and from the hands of volunteers (*n* = 124) in London UK between April 2013 and Nov 2014. Environmental sites included hotels (*n* = 100), baby care facilities (*n* = 65), handbags (*n* = 43), supermarkets (*n* = 37), restaurants (*n* = 36), public transport (*n* = 54), and a public library (*n* = 20). All specimens were plated on Mannitol Salt Agar (Oxoid, Basingstoke, UK), and then incubated aerobically at 37 °C for 24–72 h). One or two colonies for each site were selected based on staphylococci morphology [4]. The colonies were then purified on Nutrient Agar (Oxoid, Basingstoke, UK).

### Identification

All isolates were initially screened using Gram staining, catalase and coagulase tests. Those that demonstrated potential staphylococci characteristics were identified by Matrix-assisted laser desorption ionization time flight mass-spectroscopy (MALDI-TOF-MS, Microflex LT, Bruker Daltonics, Coventry, UK) in a positive linear mode (2000–20,000 m/z range) as described previously [12]. The resulting spectra were compared with reference spectra by using the Biotyper 3.0 software (Bruker Daltonics, Coventry, UK)*. Escherichia. coli* DH5α (Bruker Daltonics, Coventry, UK) was used as a standard for calibration and quality control.

### Antimicrobial susceptibility test

A panel of 11 antibiotics was used to determine the antibiotic susceptibility of all the isolates. The standard disk diffusion method was used to test AM: amoxicillin (10 μg); CEP: cefepime (30 μg); CHL: chloramphenicol (30 μg); ERY: erythromycin (5 μg); FC: fusidic acid (10 μg); GEN: gentamicin (10 μg); MUP: mupirocin (20 μg); OX: oxacillin (1 μg); PEN: penicillin (1 unit); STR: streptomycin (10 μg); TET: tetracycline (10 μg);. The susceptible, intermediate resistant or resistant were determined by the Guidelines for Susceptibility Testing [14]. The Minimum Inhibitory Concentrations (MIC) for oxacillin were additionally evaluated using “M.I.C. evaluators” (Oxoid Ltd., Basingstoke, UK).

### Detection of *mecA* gene and staphylococcal cassette chromosome *mec* (SCC*mec*) typing

The *mecA* gene was determined by using PCR method as described previously [15]. For *mecA* positive isolates, SCC*mec* types were determined by evaluating *mec* and *ccr* complexes [15].

### MLST typing of *Staphylococcus epidermidis*

Multi-locus sequence typing (MLST) was used to determine the sequence types of *S. epidermidis* [16]. Sequence types were assigned using the *S. epidermidis* database (www.mlst.net).

## Results

### Purification of isolates

A total of 643 staphylococci isolates were recovered in this study, including those from hotels (*n* = 74), baby care facilities (*n* = 46), handbags (*n* = 17), supermarkets (*n* = 89), restaurants (*n* = 96), public transportation (*n* = 94), human hands (*n* = 192) and public libraries (*n* = 35) (Additional file 1: Table S1).

### Species determination

Six hundred forty-three staphylococcal isolates belonging to 19 staphylococcal species were identified in this study. This included: *S. epidermidis* (*n* = 193), *S. hominis* (*n* = 161), *S. capitis* (*n* = 77), *S. warneri* (*n* = 63), *S. haemolyticus* (*n* = 45), *S. pasteuri* (*n* = 33), *S. saprophyticus* (*n* = 20), *S. aureus* (*n* = 12), *S. simiae* (*n* = 10), *S. cohnii* (*n *= 9), *S. sciuri* (*n* = 5), *S. pettenkoferi* (*n *= 3), *S. auricularis* (*n* = 2), *S. caprae* (*n* = 2), *S. equorum* (*n* = 2), *S. lugdunensis* (*n* = 2), *S. xylosus* (*n* = 2), *S. arlettae* (*n* = 1)*,* and *S. simulans* (*n *= 1). *S. epidermidis* was the predominant species, followed by *S. hominis*, *S. capitis*, *S. warneri*, *S. haemolyticus*, *S. pasteuri*, and *S. saprophyticus* However, the occurrence of the species varied for different sites. *S. epidermidis* was predominant among the isolates recovered from restaurants, public transport, hands and handbags, whereas *S. hominis* was predominant among the isolates recovered from supermarkets, baby care facilities and hotels and *S. haemolyticus* was predominantly isolated from the library (Table 1).

**Table 1:** Predominant and common staphylococcal species recovered from the human hands and different environmental sites

Sites	Predominant species (%)	Commonly isolated species (%)
BCF	*S. hominis* (17%)	*S. warneri* (17%)
DSH	*S. hominis* (30%)	*S. haemolyticus* (18%)
DSL	*S. haemolyticus* (29%)	*S. epidermidis* (26%)
DSR	*S. epidermidis* (38%)	*S. hominis* (35%)
DSS	*S. hominis* (44%)	*S. epidermidis* (29%)
DST	*S. epidermidis* (35%)	*S. capitis* (15%)
HB	*S. epidermidis* (40%)	*S. capitis* (27%)
HH	*S. epidermidis* (36%)	*S. hominis* (23%)

*BCF* baby care facilities, *DSH* different sites of hotels, *DSL* different sites of a library, *DSR* different sites of restaurants, *DSS* different sites of supermarkets; *DST* different sites of transportation facilities, *HB* handbags, *HH* human hand

### Antibiotic susceptibility test results

The disc diffusion method was used to test 606 isolates against a panel of 11 antibiotics. 572 (94%) isolates were resistant to at least one antibiotic, and only 34 isolates were fully susceptible. Resistance to penicillin, and fusidic acid was observed in more than 65% of all staphylococcal isolates tested. 202 (33%) isolates were resistant to streptomycin, 190 (31%) to erythromycin, 161 (27%) to amoxicillin, 98 (16%) to tetracycline, 87 (14%) to mupirocin, 59 (10%) to gentamicin, 48 (8%) cefepime, 36 (6%) oxacillin, and 21(3%) chloramphenicol (Table 2).

**Table 2:** Antibiotic susceptibility profile of staphylococci isolates recovered from general public settings

Isolates	No of isolates	Resistance to a panel of 11 antibiotics (%)										
		OX	PG	MUP	CEF	GM	FC	S	A	E	T	C
*S. epidermidis*	176	8	72	16	9	7	64	22	26	43	16	2
*S. hominis*	152	2	68	9	5	7	66	24	17	38	21	3
*S. capitis*	73	4	58	15	4	1	60	47	23	14	10	5
*S. haemolyticus*	40	10	50	13	23	15	68	73	45	0	25	10
*S. warneri*	63	3	54	17	10	22	59	51	40	27	16	2
*S. pasteuri*	31	6	69	13	6	9	69	25	31	44	17	3
*S. saprophyticus*	20	15	90	25	5	10	100	10	25	35	15	10
*S. aureus*	12	0	83	17	0	58	83	33	50	25	0	8
*S. simiae*	10	0	10	0	0	0	40	0	0	0	0	0
*S. cohnii*	9	24	67	11	33	0	78	78	22	56	11	0
*S. sciuri*	5	60	60	80	0	20	80	80	40	0	0	0
*S. pettenkoferi*	3	0	33	0	0	0	67	67	33	0	0	0
*S. lugdunensis*	2	0	50	0	0	0	50	0	0	0	0	0
*S. equorum*	2	0	50	50	0	0	50	50	50	0	50	0
*S. caprae*	2	0	100	0	100	100	100	50	50	0	0	0
*S. xylosus*	2	0	100	50	50	50	100	100	0	0	50	0
*S. auricularis*	2	0	50	0	50	0	50	0	50	0	0	0
*S. arlettae*	1	0	100	0	0	0	100	100	100	100	0	0
*S. simulans*	1	0	100	0	0	0	100	0	0	0	0	0

*OX* oxacillin (1 μg), *PG* penicillin G (1 unit), *MUP* mupirocin (20 μg), *CEF* cefepime (30 μg), *GM* gentamicin (10 μg), *FC* fusidic acid (10 μg), *S* streptomycin (10 μg), *A* amoxicillin (10 μg), *E* erythromycin (5 μg), *T* tetracycline (10 μg), *C* chloramphenicol (30 μg)

### *mecA* gene determination and SCC*mec* typing results

Sixty-eight (11%) *mecA* positive staphylococcal isolates were determined, however, no MRSA was determined in this study. *S. sciuri* had the highest *mecA* gene carriage (80%) among all 19 staphylococcal species, followed by *S. cohnii* (33%), *S. haemolyticus* (22%), and *S. saprothyticus* (20%). Other isolates demonstrated relatively lower carriage of *mecA* gene, including *S.hominis* (3%), *S.capitis* (8%), *S. epidermidis* (11%), *S.warneri* (11%), *S.pasteuri* (13%). No *mecA* gene was found in the remaining 10 species, including *S. aureus*, *S. simiae*, *S. equorum*, *S. caprae*, *S. xylosus*, *S. auricularis*, *S. simulans*, *S. arlettae S.pettenkoferi*, and *S. lugdunensis*.

SCC*mec* types were fully determined in forty-six isolates. Twenty-two out of 68 isolates lacked either the *mec* gene complex or the *ccr* gene complex. Thirteen staphylococci (19%) carried SCC*mec* type V, followed by 8 isolates carrying SCC*mec* type I (2%), 5 isolates SCC*mec* type IV (7%), 4 isolates SCC*mec* type II (6%), 1 isolate SCC*mec* type III (2%), 1 isolate SCC*mec* type VI (2%), and 1 isolate SCC*mec* type VIII (2%). In addition, three isolates harboured a new SCC*mec* type 1A, which carried combination of class A *mec* complex and *ccr* type 1. Of the ten isolates that were non-typeable, three carried a combination of class A *mec* complex and *ccrC*, six carried a combination of class B *mec* and *ccrC*, and one carried class B *mec* and *ccr* type 3 (Table 3).

**Table 3:** Molecular characterisation and antibiotic resistance of *mecA* gene positive staphylococci

ID	Sites	Species	PG	MUP	CEF	GM	FC	S	A	E	T	C	*mecA*	*mec*	*ccr*	SCC*mec*	MIC/OX (mg l^−1^)
71	HH	*S. capitis*	S	S	S	S	S	R	S	S	S	S	+	–	–	I	0.5
100	DSH	*S. cohnii*	R	R	S	S	S	S	R	R	S	S	+	Class A	5	5A	1
97	BCF	*S. cohnii*	R	S	R	S	R	R	S	R	S	S	+	Class B	1	I	**0.25**
279	HH	*S. epidermidis*	R	S	S	S	R	S	S	R	S	S	+	Class B	2	IV	2
127	DSH	*S. epidermidis*	R	S	I	S	S	S	R	R	R	S	+	Class C	5	V	2
139	DSR	*S. epidermidis*	R	R	R	S	R	S	R	R	R	S	+	Class C	5	V	2
191	DSS	*S. epidermidis*	R	S	S	S	R	S	R	S	S	S	+	Class B	4	VI	2
153	DSH	*S. epidermidis*	R	S	S	S	R	S	S	S	S	S	+	Class C	5	V	1
187	DSS	*S. epidermidis*	R	S	S	S	S	S	S	R	S	S	+	Class C	5	V	1
134	DSL	S. epidermidis*	R	S	S	S	R	R	R	R	R	S	+	Class B	1	I	1
259	HH	*S. epidermidis*	R	S	R	S	R	R	R	R	S	S	+	Class C	5	V	1
135	DSL	S. epidermidis*	R	S	S	S	R	R	R	R	R	S	+	Class B	2	IV	0.5
124	DSH	*S. epidermidis*	R	S	R	S	R	R	R	S	R	S	+	Class B	2	IV	0.5
133	DSL	S. epidermidis*	R	S	S	S	R	R	R	R	R	S	+	Class B	3	3B	0.5
126	HH	*S. epidermidis*	R	S	I	S	S	R	R	R	S	S	+	–	–	III	0.5
119	DSH	*S. epidermidis*	R	S	S	S	S	S	R	I	R	S	+	Class C	5	V	**0.12**
111	BCF	*S. epidermidis*	R	S	S	S	S	S	S	S	S	S	+	Class A	2	II	**0.12**
202	DST	*S. epidermidis*	S	R	S	S	R	I	S	S	S	S	+	Class B	5	5B	**0.12**
264	HH	*S. epidermidis*	S	R	S	S	R	R	S	S	S	S	+	Class B	2	IV	**0.06**
129	DSL	*S. epidermidis*	R	S	S	S	R	R	R	R	R	S	+	Class B	1	I	**0.03**
362	DSL	*S. haemolyticus*	R	S	I	S	S	R	R	S	S	S	+	Class C	5	V	2
367	DSL	*S. haemolyticus*	R	S	R	S	S	R	R	S	S	S	+	Class C	5	V	2
355	DSH	*S. haemolyticus*	R	S	R	R	R	R	R	S	R	S	+	Class C	5	V	2
384	HH	*S. haemolyticus*	R	R	S	S	R	R	S	S	S	S	+	Class C	5	V	2
322	DSH	*S. haemolyticus*	R	S	S	S	S	I	R	S	R	S	+	Class A	1	1A	**0.25**
382	HH	*S. haemolyticus*	R	S	I	S	R	R	S	R	S	S	+	–	–	II	**0.25**
323	DSH	*S. haemolyticus*	S	S	S	R	S	R	R	I	R	S	+	Class A	2	II	**0.12**
381	HH	*S. haemolyticus*	S	S	I	S	S	R	S	S	S	S	+	Class B	5	5B	**0.12**
360	DSH	*S. haemolyticus*	S	S	S	S	R	S	S	S	S	R	+	Class B	5	5B	**0.06**
369	DSL	*S. haemolyticus*	R	S	S	S	R	R	S	R	S	R	+	Class B	1	I	**0.03**
413	DSH	*S. hominis*	S	R	S	S	R	R	S	S	S	S	+	Class C	5	V	2
506	DSS	*S. hominis*	S	S	S	S	R	S	S	S	S	S	+	Class B	1	I	0.5
400	DSH	*S. hominis*	R	R	S	S	R	S	R	R	R	S	+	Class A	1	1A	**0.12**
326	DSH	*S. hominis*	S	S	S	S	S	S	R	I	S	S	+	Class A	1	1A	**0.06**
589	HB	*S. pasteuri*	R	S	S	S	R	R	S	R	S	S	+	Class A	5	5A	**0.25**
592	HH	*S. pasteuri*	S	R	S	S	R	R	S	S	S	S	+	Class B	5	5B	**0.25**
627	HH	*S. saprophyticus*	R	I	S	S	R	S	S	S	R	S	+	Class B	5	5B	0.5
621	DSS	*S. saprophyticus*	R	R	R	S	R	S	R	S	R	S	+	Class B	2	IV	**0.25**
630	HH	*S. sciuri*	R	R	I	S	R	R	S	S	S	S	+	Class A	4	VIII	2
632	DSH	*S. sciuri*	R	S	I	S	R	R	R	S	S	S	+	Class A	5	5A	1
633	DSH	*S. sciuri*	R	R	I	R	R	R	R	S	S	S	+	Class B	5	5B	1
629	HH	*S. sciuri*	S	R	S	S	S	S	S	S	S	S	+	–	–	II	0.25
704	HH	*S. warneri*	R	S	I	S	R	R	R	R	S	S	+	Class C	5	V	0.5
662	DSH	*S. warneri*	R	S	S	R	R	R	R	S	R	S	+	Class C	5	V	**0.25**
694	HH	*S. warneri*	R	S	S	S	S	S	S	S	R	S	+	–	–	I	**0.25**
655	BCF	*S. warneri*	S	R	S	R	R	R	S	I	S	S	+	Class B	1	I	**0.12**

Note: * *S. epidermidis* isolates with similar MLST types

*R*: resistant, *S* sensitive. *I* intermediate

*BCF* baby care facility, *DSH* different sites of hotels, *DSL* different sites of a library, *DSR* different sites of restaurants, *DSS* different sites of supermarkets, *DST* different sites of transportation facilities, *HB* handbags, *HH* human hands

*A* amoxicillin (10 μg), *CEF* cefepime (30 μg), *C* chloramphenicol (30 μg), *E* erythromycin (5 μg), *FC* fusidic acid (10 μg), *GM* gentamicin (10 μg), *MUP* mupirocin (20 μg), *OX* oxacillin (1 μg), *PG* penicillin G (1 unit), *S* streptomycin (10 μg), *T* tetracycline (10 μg)

### Multi-locus sequence typing of *S. epidermidis*

MLST was performed to determine the housekeeping genes of 13 oxacillin resistant and *mecA* positive *S. epidermidis.* MLST typing revealed that all *S. epidermidis* strains possess new MLST types. MLST types of *S. epidermidis* isolates with in house numbers of 279, 133, 134, 135, 126, 259, 124, 127, 234, 187, 308, 153 and 191 were respectively assigned as ST599, ST600, ST600, ST600, ST601, ST602, ST602, ST603, ST604, ST605, ST606, ST607 and ST608 (Table 4). Three *S. epidermidis* isolates shared the same sequence types (ST), including *S. epidermidis* 133, 134 and 135 that were isolated from different sites of a library (DSL) possessed ST600 whereas *S. epidermidis* 259, and *S. epidermidis* 124 that had ST602 sequence type were isolated from the human hands (HH) and different sites of hotels (DSH) respectively.

**Table 4:** MLST types of 13 oxacillin resistant and *mecA* positive *S. epidermidis*

ID	Sites	Species	*arcC*	*aroE*	*gtr*	*mutS*	*pyrR*	*tpiA*	*yqiL*	MLST types
279	HH	*S. epidermidis*	57	17	5	5	3	4	31	ST599
133	DSL	*S. epidermidis*	57	1	2	2	4	1	4	ST600
134	DSL	*S. epidermidis*	57	1	2	2	4	1	4	ST600
135	DSL	*S. epidermidis*	57	1	2	2	4	1	4	ST600
126	HH	*S. epidermidis*	57	25	9	5	6	1	8	ST601
259	HH	*S. epidermidis*	57	1	2	2	4	1	1	ST602
124	DSH	*S. epidermidis*	57	1	2	2	4	1	1	ST602
127	DSH	*S. epidermidis*	57	10	5	5	10	16	21	ST603
234	HB	*S. epidermidis*	57	1	1	1	2	41	1	ST604
187	DSS	*S. epidermidis*	57	1	1	2	2	1	1	ST605
308	HH	*S. epidermidis*	57	1	2	2	4	7	1	ST606
153	DSH	*S. epidermidis*	57	1	22	2	2	16	1	ST607
191	DSS	*S. epidermidis*	57	3	5	5	7	14	11	ST608

*HH* human hands, *DSL* different sites of a library, *DSH* different sites of hotels, *DSS* different sites of supermarkets

*MLST* Multi-locus sequence typing

## Discussion

### Environmental staphylococcal species

Although antibiotic resistance is commonly linked to the clinic, recent studies from different ecological niches revealed multidrug resistant bacteria is widespread in the environment [11, 12, 17].

We have previously reported on high levels of antibiotic resistance in staphylococci isolated from different environmental/public settings [11, 12]. In this study we evaluated the dissemination of antibiotic resistant staphylococci recovered from a wide range of environmental settings, and characterised the carriage of the *mecA* gene and the diversity of SCC*mec* elements in these isolates.

Six hundred and forty-three staphylococci isolates belonging to 19 species, including *S. epidermidis, S. hominis*, *S. haemolyticus*, *S. capitis*, *S. warneri, S. pasteuri, S. saprophyticus, S. cohnii, S. aureus, S. simiae, S. sciuri, S. pettenkoferi, S. lugdunensis, S. equorum, S. caprae, S. xylosus, S. auricularis, S. simulans,* and *S. arlettae*, were identified in this study. Interestingly, many of the staphylococci species recovered in our study have previously been associated with the community, preserved food, and wildlife [4, 10].

### Antibiotic resistance

**Fig. 1:**
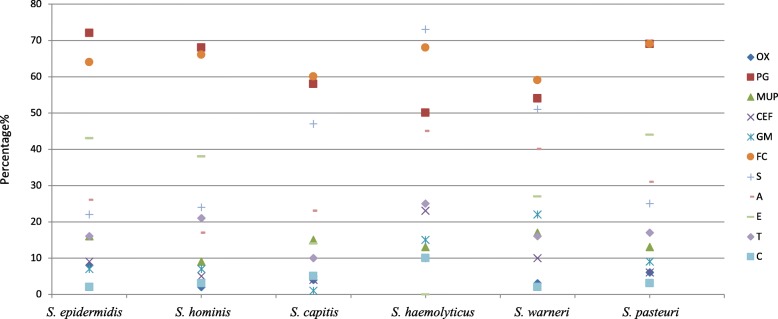
The scatter plot of staphylococcal (> 30 isolates) susceptibility profile. Legend: OX: oxacillin (1 μg); PG: penicillin G (1 unit); MUP: mupirocin (20 μg); CEF: cefepime (30 μg); GM: gentamicin (10 μg); FC: fusidic acid (10 μg); S: streptomycin (10 μg);A: amoxicillin (10 μg); E: erythromycin (5 μg); T: tetracycline (10 μg); C: chloramphenicol (30 μg)

Antibiotic resistance of staphylococci associated with healthcare settings is well documented, however, little is known about the antibiotic resistance in staphylococci isolated from different ecological niches [4]. In this study, the majority of staphylococci were resistant to penicillin (65%) and fusidic acid (66%) (Fig. 1). Despite that 80% of hospital associated CoNS (across Europe) were reported to be resistant to oxacillin [18], only 6% of CoNS were resistant to oxacillin in this study. In addition, the levels of resistance to chloramphenicol (3%), cefepime (8%), gentamicin (10%), mupirocin (14%), tetracycline (16%), and erythromycin (31%) were lower compared to those reported in clinical settings [19–22]. In contrast, the rates of resistance to fusidic acid (66%), amoxicillin (27%) and streptomycin (33%) in environmental staphylococcal isolates were higher than those reported in clinical staphylococci isolates [21, 23, 24]. It is widely accepted that higher levels of antibiotic resistance in clinical isolates are due to consistent antibiotic exposure [25]. The environment may also contribute to the development of antibiotic resistance in microorganisms due to human/ animal therapeutics, sewage, agriculture and industrial use of antibiotics [26]. Therefore, the wide dissemination of multidrug resistant CoNS in non-healthcare associated environments is a disturbing finding. In our study, 94% of staphylococcal isolates were phenotypically resistant to at least 1 antibiotic, 18% were resistant to five or more antibiotics and only 6% staphylococcal isolates were fully susceptible. The study also revealed that the number of isolates resistant to multiple antibiotics varied between the different isolation sites. The least number of multiple antibiotic resistant CoNS isolates were recovered from the public transport (58%), the highest was isolated from hotels (78%).

### Methicillin-resistant staphylococci

Methicillin resistant staphylococci pose a major public health threat, and cause severe economic and health consequences [27]. Methicillin resistance is determined by the *mecA* gene, which encodes for penicillin binding protein 2a (PBP2a) that has a low affinity to β-lactam antibiotics [28]. Hussain et al. assessed the correlation between *mecA* gene and oxacillin susceptibility breakpoints (0.5 mg l^− 1^) of 493 clinical CoNS belonging to and classified into 4 categories [29]. The *mecA* gene positive staphylococci were categorized into groups I and II, and demonstrated that group I (*S. haemolyticus* (83.3%), *S. epidermidis* (61.9%), *S. hominis* (51.8%)) differs from group II (*S. cohnii* (28.5%), *S. warneri* (27.3%), *S. saprophyticus* (9.0%)) by their high levels of *mecA*-carriage [29]. Interestingly, *S. hominis* (38%), *S. haemolyticus* (22%), and *S. epidermidis* (7%) isolated in this study harboured significantly lower levels of the *mecA* gene. Moreover, in this study *S. cohnii* (33%) and *S. saprophyticus* (10%) showed higher *mecA* gene carriage than clinical isolates reported by Hussain, et al. [29], whereas the levels of *mecA* gene carriage in *S. warneri* (6%) were lower than in clinical isolates. No *mecA* gene was detected in staphylococcal species of groups III and IV, which included *S. xylosus*, *S. lugdunensis*, *S. capitis*, *S.simulans*, and *S. schleiferi* [29]. Similarly, in this study *S. lugdunensis*, *S. xylosus* and *S. simulans* were determined to be susceptible to oxacillin and lacked *mecA* gene. However, in contrast to the reports by Hussain, et al. [29] we found that *mecA* gene was present in 8% of *S. capitis* isolates.

Oxacillin susceptible *mecA* gene positive *S. aureus* (OS-MRSA) has been reported worldwide, and the risk of induced high levels of oxacillin resistance was determined in OS-MRSA [30, 31]. In this study, 68 (46%) staphylococcal isolates were confirmed by PCR to carry the *mecA* gene, however, they were phenotypically susceptible to oxacillin with the MICs (oxacillin) varying from 0.015 to 2 mg l^− 1^. This study demonstrates the prevalence of *mecA* positive but oxacillin susceptible CoNS (OS-CoNS) in the environment. Little is known about OS-CoNS isolates recovered from the environment and their epidemiological data are limited. Additional studies are necessary to further our understanding of the prevalence and molecular epidemiology of OS-CoNS in the environment.

### SCC*mec* elements

SCC*mec* is a mobile genetic element with two essential components: the *mec* gene complex, and the cassette chromosome recombinase (*ccr*) gene complex [32]. The combination of the *mec* gene complex and *ccr* gene complex confers different SCC*mec* types [32]. SCC*mec* type I, II, III are reported to be associated with MRSA recovered from healthcare settings, whereas SCC*mec* type IV and V are mainly associated with the community [32]. Moreover, it has been shown that the size of SCC*mec* types IV and V are smaller than SCC*mec* types I, II and III, thus conferring increased mobility by their smaller size and contributing the spread of these smaller SCC*mec* elements [33] . In this study, SCC*mec* type I, II or III were found in 19% (*n* = 13) of *mecA*-positive CoNS, whereas 27% (*n* = 18) of CoNS were determined to harbour SCC*mec* type IV or V. SCC*mec* type VI and VIII were previously identified in Portugal (2006) and Canada (2009) in hospital associated MRSA (HA-MRSA) [33, 34]. In this study, we identified one of each type, however, we did not detect SCC*mec* types IX.

Becker et al., have previously summarized the community and livestock associated staphylococcal species and their SCC*mec* types, which included *S. capitis* (I, IA, II, III, IV, IVa, V, non-typeable: (NT)), *S. cohnii* (NT), *S. epidermidis* (I, IIa, IIb, III, III (variant), IV, IVa, IVb, IVc, IVd, IVe, IVg, V, VI, NT), *S. haemolyticus* (I, II, II.1, III, III (variant), IV, V, NT), *S. honomis* (I, III, IV, NT), *S. pasteuri* (IVc), *S. saprophyticus* (III, NT), *S. sciuri* (I, III, IIIA, V, VII, NT) and *S. warneri* (IV, IV.1, IVb, IVE) [4]. In this study, species associated SCC*mec* types differed and included the following: *S. capitis* (I, NT), *S. haemolyticus* (I, II, V, NT) and *S. hominis* (I, V, NT), *S. cohnii* (I, V, NT), *S. pasteuri* (NT), *S. saprophyticus* (IV, NT), *S. sciuri* (II, VIII), *S. warneri* (I, V, NT). *S. epidermidis* possessed SCC*mec* types similar to those reported previously [4].

Thirteen unclassified SCC*mec* types were determined in this study, including three carrying class A *mec* complex and *ccrC*, six had a combination of class B *mec* and *ccrC*, one carried class B *mec* and *ccr3*, and three had a combination of class A *mec* complex and *ccr* type 1. The 1A was previously defined as a new SCC*mec* type 1A by others [35]. Pseudo (ψ)-SCC*mec* harbours the *mec* complex but lacks *ccr*, while, SCC*mec*12263 is reported to carry the *ccr* complex but lacks *mec* complex [36, 37]. In this study, 21 isolates (29%) were categorized as (ψ)-SCC*mec* and SCC*mec*12263 since they lacked either *mec* complex or *ccr* complexes. ψ SCC element is characterized by lacking genes for *ccr* and *mec* [4]. One of *S. saprophyticus* isolates in this study was found to possess the ψ SCC element (Table 5).

**Table 5:** The diversity of SCC*mec* types of *mecA* gene positive staphylococci

ID	Sites	Species	PG	MUP	CEF	GM	FC	S	A	E	T	C	*mecA*	*mec*	*ccr*	SCC*mec*	MIC/OX (mg l^−1^)
75	HH	*S. capitis*	R	S	S	S	R	R	S	S	S	S	+	Class A	NT	Pseudo (ψ)-SCC*mec*	0.5
81	HH	*S. capitis*	R	S	R	S	R	R	R	R	S	S	+	NT	5	SCC*mec*12263	0.5
70	HH	*S. capitis*	R	S	S	S	S	R	S	S	R	S	+	NT	5	SCC*mec*12263	**0.25**
83	HH	*S. capitis*	S	R	S	S	R	R	S	S	S	S	+	NT	5	SCC*mec*12263	**0.12**
24	DSH	*S. capitis*	S	S	S	S	R	R	S	S	S	S	+	NT	1	SCC*mec*12263	**0.12**
108	HH	*S. cohnii*	S	S	I	S	R	R	S	R	R	S	+	Class A	NT	Pseudo (ψ)-SCC*mec*	1
308	HH	*S. epidermidis*	R	R	S	S	R	S	R	R	S	S	+	Class B	NT	Pseudo (ψ)-SCC*mec*	2
234	HB	*S. epidermidis*	S	R	S	S	R	R	S	R	R	S	+	Class A	NT	Pseudo (ψ)-SCC*mec*	1
249	DSH	*S. epidermidis*	R	S	S	S	R	R	S	S	R	S	+	NT	2	SCC*mec*12263	**0.12**
125	DSH	*S. epidermidis*	S	S	I	S	S	R	S	S	S	S	+	NT	5	SCC*mec*12263	**0.06**
185	DSS	*S. epidermidis*	R	S	S	S	S	S	S	S	S	S	+	Class C	NT	Pseudo (ψ)-SCC*mec*	**0.06**
498	DSS	*S. hominis*	R	S	S	S	R	S	S	R	S	S	+	Class A	NT	Pseudo (ψ)-SCC*mec*	0.5
426	DSH	*S. hominis*	R	S	I	S	R	R	R	R	S	S	+	Class A	NT	Pseudo (ψ)-SCC*mec*	**0.25**
412	DSH	*S. hominis*	R	S	S	S	R	S	R	R	S	S	+	NT	1	SCC*mec*12263	**0.06**
391	BCF	*S. hominis*	R	S	S	S	R	S	S	S	S	S	+	NT	5	SCC*mec*12263	**0.03**
593	HH	*S. pasteuri*	R	S	S	R	R	R	R	S	S	S	+	NT	5	SCC*mec*12263	0.5
597	HH	*S. pasteuri*	R	R	I	S	S	R	S	S	S	S	+	NT	5	SCC*mec*12263	0.5
616	BCF	*S. saprophyticus*	R	R	S	S	R	I	R	R	R	S	+	NT	5	SCC*mec*12263	256
612	BCF	*S. saprophyticus*	R	R	S	S	R	S	S	R	S	S	+	NT	NT	ψ SCC	1
659	DSH	*S. warneri*	R	R	S	S	R	R	R	S	S	S	+	NT	5	SCC*mec*12263	0.5
648	BCF	*S. warneri*	R	S	S	R	R	S	R	S	S	S	+	NT	5	SCC*mec*12263	**0.06**
645	BCF	*S. warneri*	R	S	S	S	R	S	S	S	S	S	+	NT	4	SCC*mec*12263	**0.015**

*R* resistant, *S* sensitive, *I* intermediate

*BCF* baby care facility, *DSH* different sites of hotels, *DSL* different sites of a library, *DSR* different sites of restaurants, *DSS* different sites of supermarkets; *DST* different sites of transportation facilities, *HB* handbags, *HH* human hands

*A* amoxicillin (10 μg), *CEF* cefepime (30 μg), *C* chloramphenicol (30 μg), *E* erythromycin (5 μg), *FC* fusidic acid (10 μg), *GM* gentamicin (10 μg), *MUP* mupirocin (20 μg), *OX* oxacillin (1 μg), *PG* penicillin G (1 unit), *S* streptomycin (10 μg), *T* tetracycline (10 μg)

### MLST of *S. epidermidis*

Whilst many studies have reported on the changing epidemiology of *S. aureus*, epidemiological data of other staphylococcal species are limited [38, 39]. In this study, 10 new MLST types were determined in 13 *S. epidermidis* isolates. Interestingly, although isolates recovered from human hands (*S. epidermidis* 259/ SCC*mec* V) and hotels (*S. epidermidis* 124/ SCC*mec* IV) harboured different *SCCmec* types, they shared the same MLST type ST602. In addition, three *S. epidermidis* isolates recovered from libraries (*S. epidermidis* 133, *S. epidermidis* 134, *S. epidermidis* 135) shared the same MLST type ST600 (Table 4). However, despite sharing the same MLST type *S. epidermidis* 133, *S. epidermidis* 134 and *S. epidermidis* 135 harbored SCC*mec* type 3B, I, IV respectively. Others reported that *S. epidermidis* ST2 was associated with type II, III, IV and non-typable SCC*mec*, and *S. epidermidis* ST22 harboured SCC*mec* type III, IV and V [40].

## Conclusions

Systematic analysis of staphylococci isolated from non-healthcare environments provided insights into the diversity and antibiotic susceptibility patterns of these isolates. Multi-drug resistance was commonly seen in each staphylococcal species. The prevalence of multiple antibiotic resistant staphylococci in this study provides evidence that antibiotics in the natural environments can contribute to the selection of antibiotic resistance in microorganisms. The finding of various SCC*mec* types in non-healthcare associated environments emphasizes the complexity of SCC*mec* elements. In addition to this, we also report on new MLST types that were assigned for all *S. epidermidis* isolates. This highlights the genetic variability of these isolates. In conclusion, the non-healthcare environments may act as a reservoir of multidrug resistant staphylococci, and current infection control measures are ineffective in limiting the spread of these bacteria.

## Additional file

Additional file 1: **Table S1**. Isolates collected from different environmental sites and human hands (PDF 46 kb)

## Abbreviations

AM: Amoxicillin
BCF: Baby care facility
CEP: Cefepime
CHL: Chloramphenicol
CoNS: Coagulase-negative staphylococci
DSH: Different sites of hotels
DSL: Different sites of a library
DSR: Different sites of restaurants
DSS: Different sites of supermarkets
DST: Different sites of transportation facilities
ERY: Erythromycin
FC: Fusidic acid
GEN: Gentamicin
HB: Handbags
HH: Human hands
MALDI-TOF-MS: Matrix-assisted laser desorption ionization time flight mass-spectroscopy
MIC: Minimum Inhibitory Concentrations
MLST: Multi-locus sequence typing
MR-CoNS: Methicillin resistant coagulase negative staphylococci
MRSA: Methicillin resistant *Staphylococcus aureus*
MUP: Mupirocin
OX: Oxacillin
PEN: Penicillin
SCC*mec*: Staphylococcal cassette chromosome mec
ST: Sequence types
STR: Streptomycin
TET: Tetracycline
