# Supplementary material for: The prevalence, antibiotic resistance and mecA characterization of coagulase negative staphylococci recovered from non-healthcare settings in London, UK
Source: Antimicrob Resist Infect Control. 2018 Jun 13;7:73. doi: 10.1186/s13756-018-0367-4 (PMC6000976; doi:10.1186/s13756-018-0367-4)
Supplement: Supplementary file 1 — Table S1. Isolates collected from different environmental sites and human hands (PDF 46 kb) [file 13756_2018_367_MOESM1_ESM.pdf]

Table S1. Isolates collected from different environmental sites and human hands.

Sites	DSH	BCF	DST	DSS	DSR	DSL	HB	HH
Specific sites	Toilet rims, Keyboards<br>TV remote control,<br>Toilet floor, Duvets,<br>Lift buttons, Tables,<br>Room carpet floor,<br>Water taps, Pillows,<br>Wardrobe handles,<br>Paper dispensers,<br>Bedside lights,<br>Toilet handles,<br>Basin surface,<br>Hand dryers<br>Mattresses	Mother's change bags<br>Nappy changing area<br>Child car seats<br>Dummies<br>Soft play	Pelican crossing buttons<br>Hand rails<br>Seats	Trolley handles<br>Shelves	Knife handles<br>Fork handles	Books	Handbags	Hands
No of staphylococci	74	46	94	89	96	35	17	192

Note: DSH- different sites of hotels; BCF- baby care facility; DST- different sites of transportation facilities; DSS- different sites of supermarkets; DSR- different sites of restaurants; DSL- different sites of a library; HB- handbags; HH- human hands.
